# Cortico–Cortical Connections of Primary Sensory Areas and Associated Symptoms in Migraine

**DOI:** 10.1523/ENEURO.0163-16.2016

**Published:** 2017-01-17

**Authors:** Duncan J. Hodkinson, Rosanna Veggeberg, Aaron Kucyi, Koene R. A. van Dijk, Sophie L. Wilcox, Steven J. Scrivani, Rami Burstein, Lino Becerra, David Borsook

**Affiliations:** 1 Department of Anesthesiology, Perioperative and Pain Medicine, Boston Children’s Hospital, Harvard Medical School, Boston, Massachusetts 02115; 2 Department of Neurology & Neurological Sciences, Stanford University, Palo Alto, California 94305; 3 Athinoula A. Martinos Center for Biomedical Imaging, Department of Radiology, Massachusetts General Hospital, Charlestown, Massachusetts 02129; 4 Department of Oral and Maxillofacial Surgery, Massachusetts General Hospital, Boston, Massachusetts 02115; 5 Department of Anesthesia and Critical Care, Beth Israel Deaconess Medical Center, Harvard Medical School, Boston, Massachusetts 02115

**Keywords:** connectivity, cortico–cortical, headache, migraine, pain, sensory

## Abstract

Migraine is a recurring, episodic neurological disorder characterized by headache, nausea, vomiting, and sensory disturbances. These events are thought to arise from the activation and sensitization of neurons along the trigemino–vascular pathway. From animal studies, it is known that thalamocortical projections play an important role in the transmission of nociceptive signals from the meninges to the cortex. However, little is currently known about the potential involvement of cortico–cortical feedback projections from higher-order multisensory areas and/or feedforward projections from principle primary sensory areas or subcortical structures. In a large cohort of human migraine patients (*N* = 40) and matched healthy control subjects (*N* = 40), we used resting-state intrinsic functional connectivity to examine the cortical networks associated with the three main sensory perceptual modalities of vision, audition, and somatosensation. Specifically, we sought to explore the complexity of the sensory networks as they converge and become functionally coupled in multimodal systems. We also compared self-reported retrospective migraine symptoms in the same patients, examining the prevalence of sensory symptoms across the different phases of the migraine cycle. Our results show widespread and persistent disturbances in the perceptions of multiple sensory modalities. Consistent with this observation, we discovered that primary sensory areas maintain local functional connectivity but express impaired long-range connections to higher-order association areas (including regions of the default mode and salience network). We speculate that cortico–cortical interactions are necessary for the integration of information within and across the sensory modalities and, thus, could play an important role in the initiation of migraine and/or the development of its associated symptoms.

## Significance Statement

Migraine is a multifactorial disorder that is associated with abnormalities in sensory processing, including nociceptive and non-nociceptive processing. Here we examine the cortico–cortical circuitry in the migraine brain relating to the principle primary sensory areas (vision, audition, and somatosensation). We also compare self-reported retrospective migraine symptoms in the same patients. Our results identified widespread and persistent disturbances in the perceptions of multiple sensory modalities. Furthermore, we discovered that primary sensory areas maintain local functional connectivity but express impaired long-range connections to higher-order association areas (including regions of the default mode and salience network). These findings provide new insights into the complex symptomatology of migraine and highlight the need to consider network-level cortical processes in the pathophysiology of headache disorders.

## Introduction

Cutaneous allodynia, photophobia, phonophobia, and osmophobia are clinical symptoms that accompany most migraine attacks. These sensory events may vary in intensity, as the heightened sensitivity in one sensory modality is often associated with heightened sensitivity in the other sensory modalities. The mechanisms proposed to underlie this phenomenon include the activation and sensitization of neurons along the trigeminovascular pathway (Akerman et al., 2011; Bernstein and Burstein, 2012; Pietrobon and Moskowitz, 2013; Burstein et al., 2015). Recent animal studies have shown that dura-sensitive neurons in the thalamus can respond to stimuli from more than one sensory modality (Noseda et al., 2010), and the axonal projections and termination fields of these thalamic trigeminovascular neurons appear to be widespread throughout the neocortex (Noseda et al., 2011). Such extensive inputs to diverse cortical areas may explain some of the common disturbances in neurological functions during migraine (Hodkinson et al., 2015). However, at the early cortical stage, there is the potential involvement of cortico–cortical feedback projections from higher-order multisensory areas (Hackett et al., 1998; Romanski et al., 1999; Lewis and Van Essen, 2000) and/or feedforward projections from principle primary sensory areas or subcortical structures (Falchier et al., 2002; Clavagnier et al., 2004; Cappe and Barone, 2005).

Accumulating evidence suggests that the neural basis of multisensory integration begins in early sensory processing (Ghazanfar and Schroeder, 2006). One hallmark sign of convergence is that responses elicited in primary sensory cortices by corresponding sensory inputs can be modulated by concurrently applied noncorresponding sensory inputs (Ghazanfar et al., 2005; Kayser et al., 2005, 2010; Bizley et al., 2007). Also, the primary sensory cortices do not solely respond to sensory inputs of their own principal modality (Calvert et al., 1997; Meyer et al., 2010, 2011; Liang et al., 2013). This cross-modal interaction between senses (Stein, 1998) is impaired in migraine patients but is not completely disrupted (Brighina et al., 2015). Understanding the interactions that occur when processing multiple external stimuli and activation of the trigeminal system may help to explain migraine symptoms and mechanisms by which exposure to visual, auditory, and olfactory stimuli can trigger migraine attacks. However, identifying trigger factors or premonitory features that reliably predict headache onset in migraine remains an ongoing clinical challenge (Lipton et al., 2014; Pavlovic et al., 2014).

Intrinsic functional connectivity magnetic resonance imaging (fcMRI) has emerged as a promising new tool for mapping large-scale networks in the resting human brain (Fox and Raichle, 2007). A major application of functional connectivity has been to define distinct regions of cortex (known as cortical hubs) and their corresponding networks (Buckner et al., 2013; Power et al., 2014b). In migraine and other headache disorders, this approach has been used to interrogate specific areas involved in processing pain, affect and emotion, cognition, and pain modulation (Schwedt et al., 2015). However, rather than examining sensory pathways in isolation or in disconnected regions, it has been suggested that perceptual integration is more likely to be achieved via mutual interaction of multiple regions (Ghazanfar and Schroeder, 2006; Harris and Mrsic-Flogel, 2013), thus supporting the hypothesis that the processing of sensory information arises from both local and distant cortico–cortical interactions (Sepulcre et al., 2010, 2012).

In this study, we conducted a systematic assessment of the intrinsic functional connectivity associated with the three main primary sensory areas of vision, audition, and somatosensation. Olfactory and/or chemosensory processes are not considered here because of the lack of a known primary anatomical area. However, it is noteworthy that extensive interactions are also characteristic of these sensory systems (Katz et al., 2001). Specifically, we sought to explore the complexity of the sensory networks as they converge and become functionally coupled in multimodal systems. We also assessed patients’ self-reported (retrospective) migraine symptoms, including the prevalence of sensory symptoms at different phases of the attack. We hypothesized that migraineurs would show abnormalities with respect to the networks and regions that are involved in making the complex connections between the primary sensory and higher-order distributed systems of the human brain. Such changes would provide further insights into the complex symptomatology of migraine and address the need to consider “brain-wide” network-level processes in the pathophysiology of headache disorders.

## Materials and Methods

### Ethical approval and consent

The Institutional Review Board at McLean Hospital, Harvard Medical School (Boston, MA) approved the study. All experiments fulfilled the criteria of the Helsinki accord for human research (http://www.wma.net/en/30publications/10policies/b3/). Informed written consent was obtained from all participants.

### Inclusion/exclusion criteria

All participants underwent physical and neurological examinations. Migraine patients had to meet the following criteria to be enrolled into the study: (1) had experienced episodic migraine as classified in the International Classification for Headache Disorders, Second Edition (Headache Classification Subcommittee of the International Headache Society, 2004); (2) had experienced episodic migraine for ≥3 years; and (3) had no migraine 72 h prior to the study session and no symptoms of migraine development 24 h after the scan.

A detailed medical history was taken from both patients and the control subjects. Patients were excluded if they had continuous background headache or pain, chronic migraine, or were taking daily medication including prophylactic migraine treatment. Healthy control subjects were excluded if they had any type of migraine or first-degree relatives with a history of any type of migraine. Females were also excluded if they were pregnant.

### Headache characteristics

Retrospective migraine attack characteristics were collected on study inclusion. A detailed report on the prevalence of sensory symptoms (i.e., photophobia, phonophobia, and osmophobia) was collected at different phases of the patient’s typical migraine cycle (i.e., before, during, and after). Patients were also asked to complete the Allodynia Symptom Checklist, assessing the frequency of allodynia symptoms during headache (Jakubowski et al., 2005; Ashkenazi et al., 2007; Lipton et al., 2008). The checklist responses were recorded as categorical (nominal: yes/no) variables.

### Study participants

Eighty adult right-handed participants were recruited for the study (mean ± SD age, 32.7 ± 9 years; age range, 18–50 years). The cohort included 40 patients who experienced episodic migraine and 40 individually age-matched (±1 year) and sex-matched healthy control subjects (10 males/30 females). Migraine patients reported a mean migraine (disease) duration of 15 ± 9 years (range, 3–39 years). The attack frequency was recorded as number of episodes per month (6.9 ± 5 attacks/month). Most patients reported migraine without aura (*N* = 24), but some reported migraine with aura (*N* = 16). Laterality of headache pain was reported as either unilateral (*N* = 10 right-sided, *N* = 10 left-sided) or bilateral (*N* = 20). The participants were studied intensively using a range of neuroimaging and behavioral tests, and some of these data have been used to address distinct questions previously (Hodkinson et al., 2016).

### MRI acquisition

Participants were scanned in the supine position on a 3 T Siemens whole-body MRI scanner with a standard 12-channel head coil. A high-resolution T1-weighted anatomical scan was acquired using a 3D magnetization-prepared rapid gradient echo sequence (TI, 1100 ms; TR, 2000 ms; TE, 3.5 ms; flip angle, 8°; FOV, 256 mm^2^; matrix, 256 × 256; 224 slices; voxel size, 1 × 1 × 1 mm). Resting-state fcMRI (rs-fcMRI) data were acquired using a gradient-echo–echoplanar imaging (EPI) sequence [TE, 30 ms; TR, 2010 ms; flip angle, 90°; FOV, 224 mm^2^; matrix, 64 × 64; number slices, 34; slice thickness, 4 mm (no gap); voxel size, 3.5 × 3.5 mm^2^; number of volumes, 300; total scan time, 10 min and 5 s]. Slices were acquired in interleaved ascending order, parallel to the anterior commissure–posterior commissure line. All participants were instructed to stay awake, keep their eyes open, and minimize head movement.

### Imaging software

All imaging data were preprocessed using SPM12 (http://www.fil.ion.ucl.ac.uk/spm) and customized scripts written in MATLAB version R2015a (MathWorks). Visualization of the brain data was performed using CARET software (Computerized Anatomical Reconstruction Toolkit, version 5.65) and the PALS (population average, landmark- and surface-based) surface (Van Essen et al., 2001; Van Essen, 2005). Additional statistical analyses were performed in R version 3.1.2 (R Foundation for Statistical Computing).

### Image preprocessing

Resting-state fcMRI data were preprocessed in accordance with previously described procedures (Van Dijk et al., 2010, 2012; Power et al., 2012). The steps involved in the pipeline included the following: dropping volumes (the first four volumes of each run were discarded to allow for T1 equilibration effects); slice timing correction (compensation for slice-dependent time shifts were corrected per volume); motion correction (rigid body translation and rotation from each volume to the first volume were used to correct for head motion); spatial normalization [normalization was achieved by computing affine and nonlinear transforms of the mean motion-corrected image to the Montreal Neurological Institute BOLD EPI template]; spatial smoothing (data were resampled to 2 mm isotropic voxels and spatially smoothed using a 6 mm full-width at half-maximum Gaussian kernel); motion and physiological regression [reduction of spurious or regionally nonspecific variance was removed by regression of 18 nuisance variables, including six parameters obtained by rigid body head motion correction, with the signal averaged over the whole brain (global signal), the lateral ventricles (CSF), and the white matter, and the first temporal derivative of each regressor also included to account for temporal shifts in the BOLD signal]; and temporal filtering (bandpass filtering was performed with a passband of 0.01–0.08 Hz). Filtering was performed by a Butterworth filter with a specified filter order of 4.

### Quality assurance and motion scrubbing

Particular care was taken to minimize the impact of head motion on the fcMRI correlations. As described previously (Van Dijk et al., 2012), we evaluated in-scanner head motion using the realignment parameters from the SPM Realignment routine. This estimation derives a motion transformation matrix for each time point, including three translations and three rotations. For each individual, the data were passed through a procedure that detects the framewise displacement (FD) of the head from one volume to the next (Power et al., 2012). Frames with a motion level >0.5 mm/TR rotations >1° or BOLD signal changes >2 SDs were scrubbed from the data. This “scrubbing” procedure used temporal masks to remove motion-contaminated data from regression and correlation calculations by excising unwanted data and concatenating the remaining data. The fraction of frames excluded did not exceed 15% of the total number of volumes (i.e., 85% sample retention). We also used measurements of absolute displacement (AD) of the head from the origin position at every time point, including total movement (maximum difference in position in millimeters) and total head rotation (maximum difference in rotation in degrees; Van Dijk et al., 2012). The purpose of these absolute measures is to index head movement, not to precisely model it. As expected, patients with migraine did not have significantly more head motion than healthy control subjects (Table 1).

### Seed-based analysis

Six seed regions were chosen to represent networks of the following primary sensory modalities: vision, audition, and somatosensation. The coordinates for these areas were as follows: primary visual cortex (V1): left (−14, −78, 8); right (10, −78, 8); 6 mm spheres; primary auditory cortex (A1): left (−54, −14, 8), right (58, −14, 8), 6 mm spheres; primary somatosensory cortex (S1): left (−59, −16, 41); right (59, −16, 41), 4 mm spheres.

Coordinates for the visual and auditory cortices were taken directly from the literature (Sepulcre et al., 2012). Somatosensory localization of S1 was selected based on the coordinates of an fMRI study involving mechanical stimulation of the forehead representing the ophthalmic trigeminal division, V1 (Moulton et al., 2009). This region was chosen because migraine pain is restricted to the head, often affecting the periorbital area and the eye (Barmettler et al., 2015).

### Boundaries of cortical association networks

Given our hypothesized role of higher-order cortical processes in the pathophysiology of headache disorders, we sought to relate our seed-based analysis with an independent functional atlas derived from a clustering approach (http://freesurfer.net/fswiki/CorticalParcellation_Yeo2011; Yeo et al., 2011). To quantify changes in specific functional networks, we used the seven-network parcellation scheme provided by Yeo et al. (2011), which is based on data from 1000 healthy adults. The boundaries of these seven networks were projected onto the MI152 template (see Fig. 7). Groupwise connectivity values were then averaged within each network.

### Hierarchical statistical model and contrasts

Pearson correlation coefficient (*r*) maps were computed between the mean time course from each of the six seed regions and all other brain voxels. The individual correlation maps were converted to *z*-maps using Fisher’s *r-*to*-z* transformation. Group-level statistical comparisons were computed under the framework of the general linear model using a random-effects flexible factorial ANOVA. In this model, the individual Fisher *z*-transformed correlation maps were the repeated-measures with factors for subject and group. This allowed us to take into account both the differences in variances between the groups and differences (within-subject) across sensory domains of the individual seed maps. This framework has the important advantage of retaining the intrinsic spatial and temporal characteristics of the data without the need for averaging multiple time courses from each seed region or performing dimensionality reduction procedures (Cole et al., 2010). Contrast specification was designed to examine the increasing complexity of the sensory networks as they converge and become functionally coupled in polymodal systems. Each contrast incorporated the left and right seeds from the primary sensory areas, which were combined as either unimodal (V1, A1, S1), bimodal (V1/A1, V1/S1, A1/S1), or multimodal (V1/A1/S1). Significant clusters were displayed with a probability threshold of *p* < 0.05, corrected for multiple comparisons using familywise error (FWE) rate.

## Results

### Migraine symptom profile

#### Photophobia, phonophobia, and osmophobia

**Figure 1.**
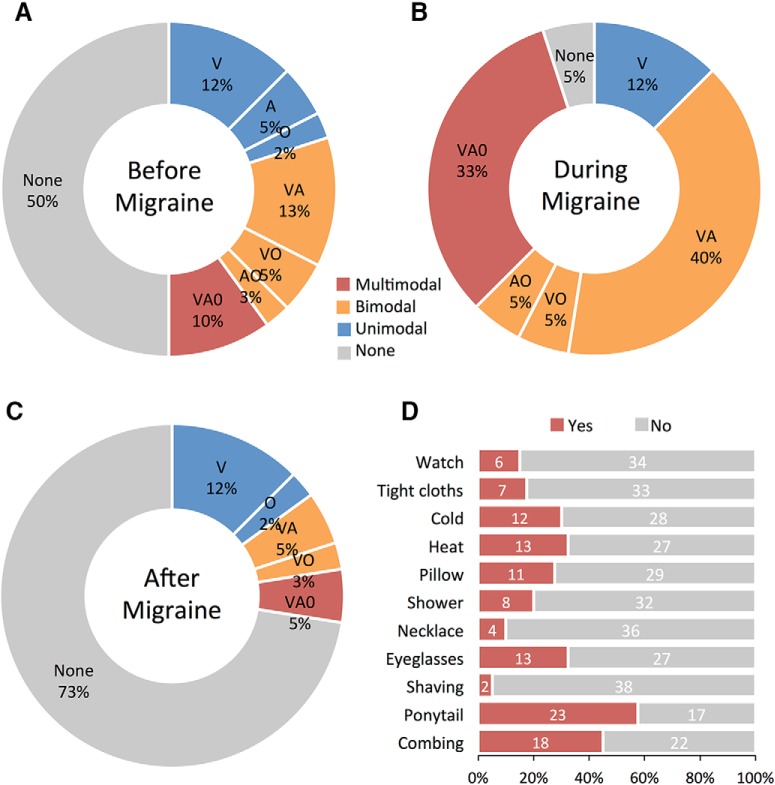
Migraine sensory symptom profile. ***A–C***, Total proportion of unimodal, bimodal, and multimodal sensory abnormalities reported before, during, and after migraine attacks (Doughnut plots). V, Photophobia; A, phonophobia; O, osmophobia). ***D***, Total proportion of positive responses to individual questionnaire items of skin hypersensitivity. Histogram shows the percentage of patients that positively reported symptoms, and those that were unaware of any abnormal skin sensitivity (Jakubowski et al., 2005; Ashkenazi et al., 2007; Lipton et al., 2008).

The participants were asked to describe whether they experience any sensory abnormalities before, during, and after migraines (Fig. 1*A–C*). A total of 50% of the patients reported sensory changes before their migraine (19% unimodal, 21% bimodal, 10% multimodal), whereas 50% were unaware of any sensory changes. These proportions changed to 95% with symptoms (12% unimodal, 50% bimodal, 33% multimodal) and 5% without symptoms during the migraine attack. Continuing through to the postdrome, 27% of patients reported at least one type of sensory change after their migraine (14% unimodal, 8% bimodal, 5% multimodal), and 73% were unaware of any sensory changes. The proportion of bimodal and multimodal sensory abnormalities was constantly high across all phases of the migraine attack.

#### Cutaneous allodynia

Subjects were also asked to fill out a questionnaire to determine whether cutaneous allodynia usually developed during their migraine attack (Fig. 1*D*). A total of 87% of the patients reported at least one type of skin hypersensitivity during a migraine attack, and 13% reported they were unaware of any abnormal skin sensitivity. These proportions changed to 55% reporting a minimum of three or more symptoms. The total number of positive responses to the individual questions varied greatly. Certain items in the questionnaire were sex specific (e.g., shaving, earrings), some items were not applicable to every patient (e.g., eyeglasses), and not every patient was able to reflect on whether a certain activity was bothersome during migraine (declared unsure).

### Unimodal networks

#### Visual cortex

**Figure 2.**
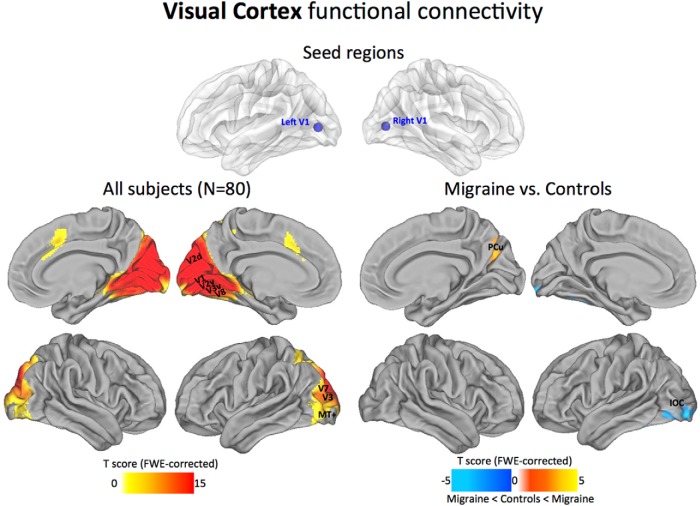
Visual cortex functional connectivity. The statistical maps illustrate the direct connectivity of the visual cortex across all subjects in the sample (*N* = 80; left). Groupwise changes in functional connectivity (migraine vs control subjects) are displayed on the right. All statistical images are displayed with a cluster probability threshold of *p* < 0.05, corrected for multiple comparisons (FWE). Seed regions used to generate the contrasts are projected onto glass brains for reference purposes. Data are shown in Caret PALS space, with multiple views of the left/right hemispheres. V1, V2, V3, V7, V8, MT+, Visuotopic areas.

Across all subjects, from the early visual seeds in V1/Brodmann area (BA) 17, we found functional connections to the main visuotopic areas: V1, V2, V3, V7, V8, and MT+. Reference boundaries for visuotopic-mapped areas are based primarily on fMRI studies of human retinotopic mapping (Hadjikhani et al., 1998; Van Essen, 2005). In migraine patients compared with control subjects, the visual networks displayed reduced anticorrelation to a small region of the precuneus (PCu) and decreased positive correlations to an area of the inferior occipital cortex (IOC)/middle occipital cortex (Fig. 2).

#### Auditory cortex

**Figure 3.**
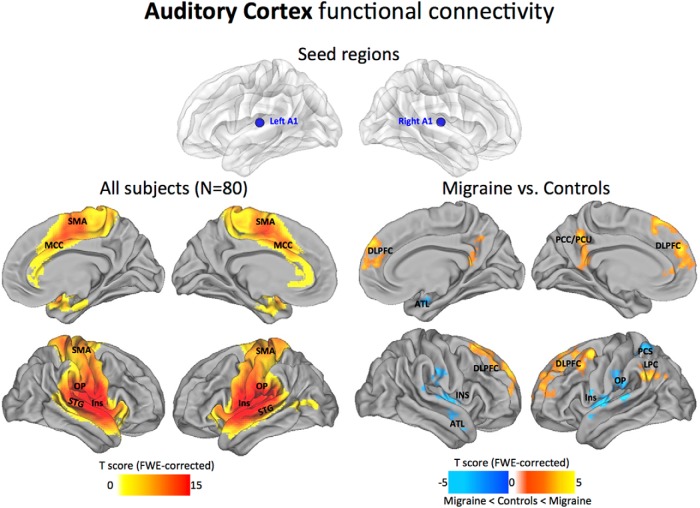
Auditory cortex functional connectivity. The statistical maps illustrate the direct connectivity of the auditory cortex across all subjects in the sample (*N* = 80; left). Groupwise changes in functional connectivity (migraine vs control subjects) are displayed on the right. All statistical images are displayed with a cluster probability threshold of *p* < 0.05, corrected for multiple comparisons (FWE). Seed regions used to generate the contrasts are projected onto glass brains for reference purposes. Data are shown in Caret PALS space, with multiple views of the left/right hemispheres.

Across all subjects, the A1 seed locations showed dense connections within local auditory-related regions (including the belt and parabelt), strong connections with operculum (OP), insula (Ins), superiortemporal gyrus (STG), and, to a lesser extent, the sensorimotor area (SMA), and mid-cingulate cortex (MCC). In the migraine patients compared with control subjects, the auditory networks displayed reduced anticorrelations to regions of the prefrontal cortex [specifically, dorsal lateral prefrontal cortex (DLPFC), PCu, and posterior cingulate cortex (PCC)], and the lateral parietal cortex (LPC). Decreased positive correlations were found to the Ins and OP cortex, posterior central sulcus (PCS), and regions of the anterior temporal lobe (ATL; Fig. 3).

#### Somatosensory cortex

**Figure 4.**
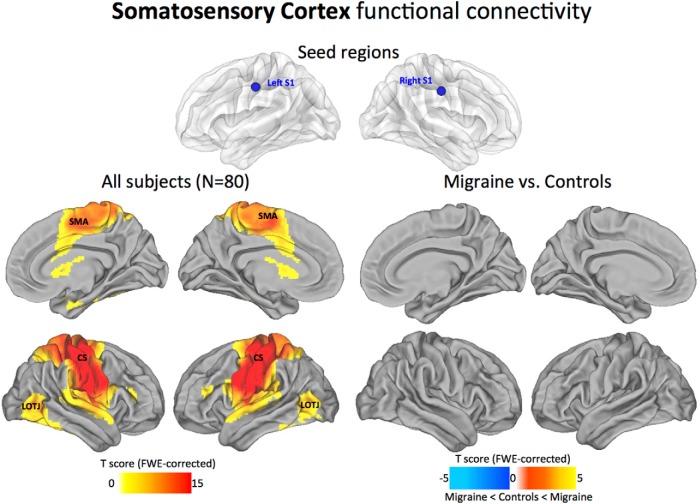
Somatosensory cortex functional connectivity. The statistical maps illustrate the direct connectivity of the somatosensory cortex across all subjects in the sample (*N* = 80; left). Groupwise changes in functional connectivity (migraine vs control subjects) are displayed on the right. All statistical images are displayed with a cluster probability threshold of *p* < 0.05, corrected for multiple comparisons (FWE). Seed regions used to generate the contrasts are projected onto glass brains for reference purposes. Data are shown in Caret PALS space, with multiple views of the left/right hemispheres.

Across all subjects, the S1 periorbital seed locations showed dense connections along the entire somatomotor cortex, with the primary motor and somatosensory cortices interlocked by mutual connections across the central sulcus (CS). Weaker connections were present to the lateral occipitotemporal junction (LOTJ). In the migraine patients compared with control subjects, the somatosensory networks displayed no significant changes in positive or negative correlations between the groups (Fig. 4).

### Bimodal networks

**Figure 5.**
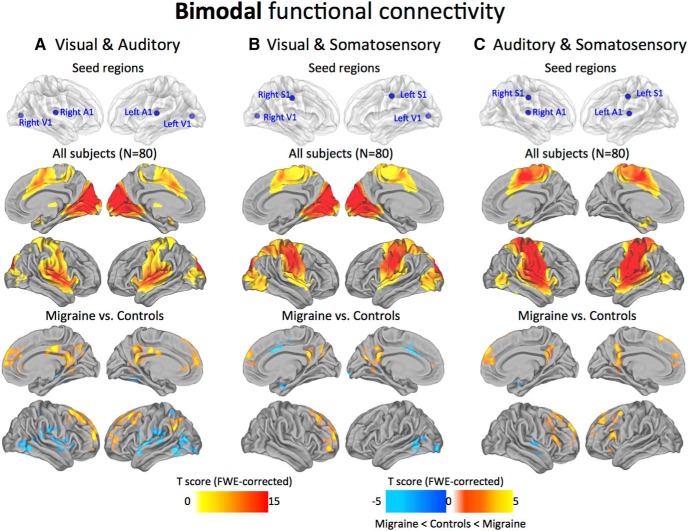
Bimodal functional connectivity. Seed regions used to generate the contrasts are projected onto glass brains for reference purposes (top row). The statistical maps illustrate the connections among visual, auditory, and somatosensory cortices across all subjects in the sample (*N* = 80; middle row). Groupwise changes in functional connectivity (migraine vs control subjects) are displayed on the bottom row. All statistical images are displayed with a cluster probability threshold of *p* < 0.05, corrected for multiple comparisons (FWE). Data are shown in Caret PALS space, with multiple views of the left/right hemispheres.

Topographically, the functional connectivity patterns for the combined pairs of sensory modalities were remarkably similar to their respective unimodal maps. However, the comparison between migraine patients and control subjects revealed some common features that appeared to converge across all the possible pairs. Specifically, the migraineurs demonstrated reduced anticorrelations with midline medial prefrontal and parietal areas (i.e., PCC/PCu, mPFC/DLPFC), and the LPC. Less consistent decreases in positive correlations were observed to the MCC, LOTJ, Ins, and OP cortex (Fig. 5).

### Multimodal networks

**Figure 6.**
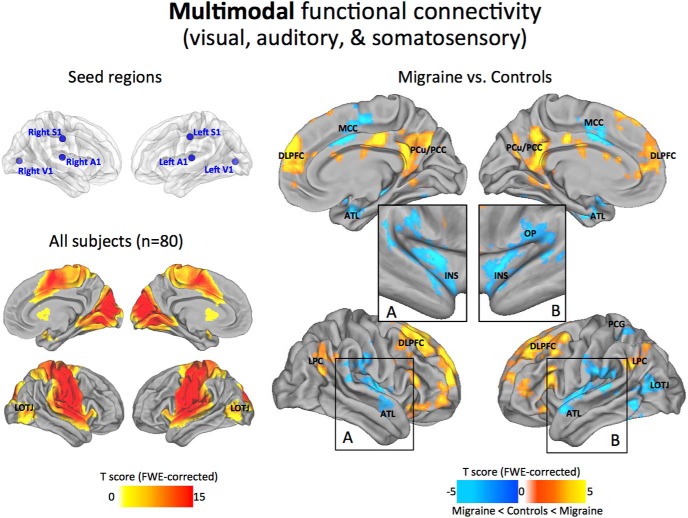
Multimodal functional connectivity. Seed regions used to generate the contrasts are projected onto glass brains for reference purposes (top left). The statistical maps illustrate the connections among all three sensory cortices across all subjects in the sample (*N* = 80; bottom left). Groupwise changes in functional connectivity (migraine vs control subjects) are displayed on the right. To better visualize the INS-OP region we also show the results as inflated projections (inset boxes in ***A*** and ***B***). All statistical images are displayed with a cluster probability threshold of *p* < 0.05, corrected for multiple comparisons (FWE). Data are shown in Caret PALS space, with multiple views of the left/right hemispheres.

Not surprisingly, the functional connectivity pattern from the three sensory modalities was comparable to the unimodal and bimodal maps. However, it is noteworthy that any inconsistencies in the bimodal networks appear to have stabilized in the vicinity of the LOTJ, reflecting a possible common point of convergence among all three sensory modalities (Beauchamp, 2005). Following comparisons between the migraineurs and control subjects, we revealed a much more defined set of regions than that described in the previous unimodal or bimodal analysis. Specifically, migraineurs demonstrated reduced anticorrelations to distinct cortical regions of the PCC/PCu, mPFC/DLPFC, and LPC, best known as the cortical hubs of the default-mode network (DMN). In the opposite direction, migraineurs showed decreased positive correlations to areas of the MCC and operculoinsular cortex, which have been described as key hubs of the ventral attention/salience network. In addition, the correlation maps showed reduced connectivity to the left LOTJ, left postcentral gyrus (PCG), and ventral/dorsal regions of the ATL (Fig. 6).

### Relationship between primary sensory areas and higher-order association networks

**Figure 7.**
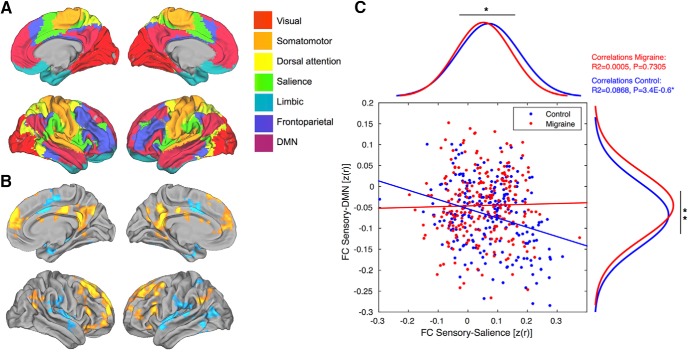
Relationship between primary sensory areas and higher-order associative networks. ***A***, Large-scale seven-network parcellation of the human cerebral cortex based on 1000 subjects (top left). ***B***, Groupwise changes in functional connectivity between migraine and control groups (bottom left). ***C***, Scatter plots and two marginal histograms showing the differences in correlated and anticorrelated spontaneous activity of DMN and salience networks. Both groups are normally distributed and significant after two-tailed *t* tests. *p < 0.05, **p < 0.005. Linear trends based on Pearson’s linear correlation coefficients (*R^2^* and *p* values are displayed in the top-right corner).

In parallel to the seed-based analyses described above, we separated the cortex into seven large-scale functional networks to examine the integrity of connections between the primary sensory and association areas (Fig. 7*A*,*B*). The amplitude of connectivity values indicates that both groups are normally distributed, and significantly different after paired *t* tests (sensory-salience: *p* = 0.0214, *t* = 2.3156; sensory-DMN: *p* = 0.0015, *t* = −3.2114). Also, the functional coupling between the correlated and anticorrelated spontaneous activity in DMN and salience networks was impaired in patients with migraine (Pearson’s correlation: control subjects: *R* ^2^ = 0.0868, *p* = 3.4E-06; migraine patients: *R* ^2^ = 0.0005, *p* = 0.7305; Fig. 7*C*).

## Discussion

The use of resting-state intrinsic functional connectivity enabled us to examine the cortico–cortical connections in the migraine brain relating to the principle sensory modalities (vision, audition, and somatosensation). These primary systems are recognized as essential cerebral areas for early sensory processing and thus could underlie important pathophysiological processes involved in sensory integration. We discuss the clinical features of migraine and their possible relationship to the disruptions in cortico–cortical interactions.

### Disturbances of multiple sensory modalities in migraine

As expected, the migraine patients reported a high incidence of sensory abnormalities (Fig. 1). Photophobia was the most commonly reported symptom, but the occurrence of phonophobia and/or osmophobia was not uncommon. Of particular interest is the relative proportion of sensory symptoms in the premonitory period continuing through to the postdrome. Nonheadache symptoms are well documented in the premonitory phase of migraine (Giffin et al., 2003), and these findings are consistent with those of other studies indicating that hypersensitivity to sensory stimuli (likely due to central sensitization) is an early phenomenon in the process of a migraine attack (Burstein et al., 2004). Further evidence of central sensitization in the trigeminal–cortical pathway (Burstein et al., 1998) was revealed through the presence of expanding cutaneous allodynia (CA) and hyperalgesia (Burstein et al., 2010). Somatosensory hypersensitivity and the development of CA symptoms occurs in approximately two-thirds of migraineurs during an attack (Ashkenazi et al., 2007; Bigal et al., 2008; Lipton et al., 2008), and approximately the same distribution of CA was reported in our cohort of patients (i.e., 87% or 55% reporting one or three or more symptoms, respectively). Together, these clinical features of migraine are consistent with abnormalities in sensory processing, including central sensitization in the trigeminocortical system. The persistent occurrence of photophobia, phonophobia, and/or osmophobia in conjunction with CA (all prominent features of migraine) suggests a common underlying disease pathway involving multisensory integration. This hypothesis is supported by recent evidence showing that cross-modal interaction between senses (Stein, 1998) is impaired in migraine patients (Brighina et al., 2015).

### Local connectivity profiles of primary sensory areas

Early sensory cortical areas are examples of areas with predominantly local hierarchical connections (Felleman and Van Essen, 1991; Sepulcre et al., 2010). In accordance with this view, our maps show consistently high levels of local connectivity across the sensory areas, with the general topography of the seed regions closely tracking the estimated boundaries of the principle cortices (Sepulcre et al., 2010; Yeo et al., 2011). Within the visual cortex, the V1 seed region showed dense local connectivity across the main visuotopic areas (V1, V2, V3, V7, V8, and MT+; Fig. 2). This observation aligns with both macaque anatomy and estimated human areal boundaries of retinotopically mapped visual areas (Hadjikhani et al., 1998). Regions at or near primary somatosensory and auditory cortices also displayed high levels of local connectivity. In particular, dense local connectivity was observed across BAs 43, 42, and 41 in the auditory cortex, with similar mutual interlocking connections along the entire length of the central sulcus of the somatosensory cortex. Interestingly, we found that the maps possessing multimodal sensory connectivity displayed strong connections with the LOTJ, which might reflect a common point of convergence among all three sensory modalities (Beauchamp, 2005; Fig. 6). Collectively, our analysis shows that local functional connectivity in the primary sensory areas remains intact and appears unaffected by the migraine condition.

### Impaired coupling between primary sensory and higher-order associative networks

In addition to local connectivity profiles, intrinsic functional coupling provides information about long-range between-network interactions. Our results confirm that the changes associated with the sensory systems may affect both correlated and anticorrelated spontaneous activity, causing a shift in the dynamic interplay between two large-scale networks representing opposing components of brain function (Fox et al., 2005; Fig. 7). Considering the relevance of these networks, they have become known as the default mode and ventral attention/salience networks, with multiple hubs capable of long-distance cortico–cortical interactions (Achard et al., 2006; Buckner et al., 2009). While we cannot describe these changes in the context of an activated brain state during migraine, it has been proposed that the salience network represents a basic system through which significant salient events are detected, incorporating afferent information from a variety of somatic and visceral sensory modalities (Craig, 2002, 2003; Iannetti and Mouraux, 2010; Critchley and Harrison, 2013). Although no consensus on the function of the default network has been reached, one frequent observation is that this network increases its activity during passive/resting brain states, suggesting a potential role in internal cognitive processes as contrast to stimulus-based perceptions (Andrews-Hanna et al., 2010). Similar features of intrinsic functional connectivity, including deficits relating to the DMN, have been observed in other chronic pain conditions, such as chronic back pain, complex regional pain syndrome, osteoarthritis, and temporomandibular disorder (Loggia et al., 2013; Baliki et al., 2014; Kucyi et al., 2014). Patients with functional pain disorders often complain of generalized sensory hypersensitivity, finding sounds, smells, or even everyday light aversive. Therefore, it is possible that deficits in sensory-processing pathways are related to hypersensitivity across the chronic pain population (Pujol et al., 2014).

### Limitations, challenges, and future perspectives

The present work has several potential limitations that are worth considering. First, we were unable to stratify patients according to their clinical symptomatology. This was because the numbers of patients within each of the possible subgroups (i.e., unimodal, bimodal, and multimodal) was much too small; hence, we were unable to assess the direct relationship between functional connectivity and individual differences in the degree or quality of the sensory abnormalities. This interaction may be explored in more detail by future studies to better understand the role of different brain areas in predicting specific phenotypes of migraine (Cucchiara et al., 2015). Second, the impact of head motion can systematically alter correlations in resting state functional connectivity. For example, it has been shown that head movements can produce spurious but structured noise in resting-state scans, thus causing distant-dependent changes in signal correlations (Power et al., 2012; Satterthwaite et al., 2012; Van Dijk et al., 2012). To address this problem, several groups have found a benefit of censoring high-motion data (Power et al., 2012; Satterthwaite et al., 2013; Yan et al., 2013a). Accordingly, we used a similar within-subject, censoring-based artifact removal strategy based on volume censoring, which should help to reduce group differences due to motion (Power et al., 2014a). In addition, we performed a systematic assessment of motion-related differences across subjects but found no significant differences between the groups quality assurance parameters (Table 1). Until better motion correction strategies are devised, censoring remains a useful tool for reducing or eliminating motion-related variance in resting-state fMRI data (Power et al., 2015). Third, we acknowledge that global signal regression (GSR) remains a controversial rs-fcMRI data-processing step, particularly for interpreting subsequent anticorrelations (Fox et al., 2009; Murphy et al., 2009; Carbonell et al., 2011; Chai et al., 2012; Keller et al., 2013). The use of GSR in this article was motivated by evidence that it can improve spatial specificity (Fox et al., 2009), correspondence to anatomy (Fox et al., 2009), and agreement with electrophysiology (Keller et al., 2013). Several studies have also shown that regressing the global signal is effective at removing motion artifacts (Satterthwaite et al., 2013; Yan et al., 2013a,b; Power et al., 2014a). However, it is important to note that when rs-fcMRI data are processed with GSR, anticorrelations are effectively mathematically partial correlations controlling for widely shared variance, leading to debate over the appropriate interpretation of observed anticorrelations (Fox et al., 2009; Murphy et al., 2009; Weissenbacher et al., 2009; Carbonell et al., 2011; Chai et al., 2012; Saad et al., 2012; Keller et al., 2013). As the field continues to debate the use of GSR, we remain open as to how best to interpret the changes in anticorrelations.

**Table 1:** Quality assurance and motion scrubbing

Parameters	Migraine	Control subjects	*p* value
FD			
Mean movement (mm)	0.095 (0.05)	0.079 (0.04)	0.131
Number of movements	10.85 (5.8)	10.75 (4.2)	0.931
AD			
Total movement (mm)	1.484 (1.6)	0.997 (0.7)	0.097
Total rotation (degrees)	0.025 (0.02)	0.018 (0.01)	0.151

Data show the mean (±SD) with associated *p* value following two-tailed *t* test. Mean movement, Average change in position between TRs; number of movements, number of motion spikes in the timeseries; total movement, maximum difference in position in millimeters; total rotation, maximum difference in rotation in degrees.

### Conclusion

In summary, we have shown widespread and persistent disturbances in the perceptions of multiple sensory modalities, which is compatible with the clinical presentation of migraine in the general population. Consistent with these observations, we found that primary sensory areas maintain local functional connections but express impaired long-range connectivity to higher-order association areas (including regions of the default mode and salience network). On the basis of this collective evidence, we propose that cortico–cortical interactions are necessary for the integration of information within and across the sensory modalities and thus could play an important role in the initiation of migraine and/or the development of its associated symptoms.

## Synthesis

Reviewing Editor: Mary Heinricher, Oregon Health & Science University Decisions are customarily a result of the Reviewing Editor and the peer reviewers coming together and discussing their recommendations until a consensus is reached. When revisions are invited, a fact-based synthesis statement explaining their decision and outlining what is needed to prepare a revision will be listed below. The following reviewer(s) agreed to reveal their identity: Katherine Martucci, Marwan N. Baliki.

In this study, the authors used resting state fMRI to examined changes in intrinsic functional connectivity in subjects suffering from migraine associated with sensory abnormalities (photophobia, phonophobia, and osmophobia) compared to healthy controls. Specifically, they explored and contrasted the effects of convergence and interactions of the primary sensory systems, including vision, audition, and somatosensation, with brain regions thought to be involved in perception and information integration, the default mode network (DMN) and the saliency network (SN). The authors also assessed reported migraine symptoms, including the prevalence of sensory symptoms at different phases of the migraine attack. In an additional analysis, the authors investigated the extent of correlation between the sensory networks and the DMN and SN networks. They found that sensory networks in subjects with migraine exhibited significantly decreased anti-correlation with the DMN and decreased correlation with SN. The authors concluded that migraineurs are characterized by abnormal functional connectivity between networks involved in linking external and internal information.

The manuscript is a nice contribution to the understanding of what is going in the brains of individuals suffering from migraine headache. The data collection and analyses appear to be well conducted and thorough. Indeed, the authors should be commended for their care in the analysis of data inducing controlling for noise and motion artifacts and appropriate rigorous statistical designs. Figure 7 is particularly compelling, and provides a wonderful visual summary of the main results. Nevertheless, some aspects of how the manuscript and results are put together could be improved by additional thought and possibly additional analysis.

A primary concern is the absence of any association between the changes in functional connectivity and sensory symptoms. This association is laid out as one of the main aims of the paper. While the authors show functional connectivity differences across all modalities, they do not relate it to the prevalence of multi-sensory symptoms observed in the subjects, which makes the functional connectivity findings difficult to interpret. As one example, are their specific connectivity changes in subjects with bi-modal symptoms compared to those with uni- or multi-modal symptoms? An analysis of the association between behavioral symptoms and brain connectivity would greatly enhance the paper, and should be doable.

In some ways, the underlying arguments are oddly developed and difficult to follow. First, the manuscript includes a significant much focus on sensory, auditory, and visual seeds/networks, but then ends with conclusions about the DMN and salience networks as the impact statement. The conclusion therefore seems highly disjointed and disconnected from the rest of the manuscript, and not where the reader is lead to believe it is heading. Rethinking and rewriting this would certainly help readers understand the significance of the work.

The emphasis on “multisensory” networks and their integration can also be confusing. The study as conducted actually focuses on “multiple” sensory networks, rather than multisensory integration. That is, most neuroscientists would not say that V1+A1+S1 = multisensory. Again, this could remedied by rewriting (substituting “multiple sensory” for “multisensory” throughout the manuscript). Alternatively, and this might make the manuscript more impactful (and better match the stated hypotheses), actually extracting and analyzing data from multisensory networks based on multisensory seed regions.

It would be most novel and interesting if the authors could somehow integrate the pre/post migraine data that they collected. They state that the limited n precludes statistical reliability, but if there were a way to integrate these symptom data with the neuroimaging findings, it would help to advance the field for the next, sufficiently powered, study.

Some additional specific comments.

1. On page 16, it is somewhat unclear what is being done for the relationship between networks and groups and why these analyses are being conducted. Are there specific hypotheses? Do these results reflect decreased counterbalancing of salience and DMN networks that has been shown before in several disease states?

2. The reasoning behind excluding participants who had recently experienced a migraine or were developing a migraine. (page 6, line 114, iii) would be enlightening.

3. Is there information (either from the authors' own work or the literature) on how consistent pre- and post-migraine symptoms are? That is, for a given individual, are these consistent for different episodes.

4. Page 18 line 354, “suggests” might be more appropriate than “confirms”.
